# Trauma Patient Volume and the Quality of Care: A Scoping Review

**DOI:** 10.3390/jcm12165317

**Published:** 2023-08-15

**Authors:** Wouter Foppen, Yvette Claassen, Debby Falck, Nardo J. M. van der Meer

**Affiliations:** 1 Department of Radiology and Nuclear Medicine, University Medical Center Utrecht, Utrecht University, 3584 CS Utrecht, The Netherlands; 2 Department of Surgery, Leids Universitair Medisch Centrum, 2333 ZA Leiden, The Netherlands; 3 Department of Neurology, HagaZiekenhuis, 2545 AA The Hague, The Netherlands; 4 Department of Medicine, Catharina Hospital, 5623 EJ Eindhoven, The Netherlands; 5 TIAS School for Business and Society, 5037 AB Tilburg, The Netherlands

**Keywords:** trauma systems, polytrauma, outcome analysis, mortality, quality of healthcare

## Abstract

Background: Healthcare stakeholders in the Netherlands came to an agreement in 2022 to deal with present and future challenges in healthcare. Among others, this agreement contains clear statements regarding the concentration of trauma patients, including the minimal required number of annual severe trauma patients for Major Trauma Centers. This review investigates the effects of trauma patient volumes on several domains of the quality of healthcare. Methods: PubMed was searched; studies published during the last 10 years reporting quantitative data on trauma patient volume and quality of healthcare were included. Results were summarized and categorized into the quality domains of healthcare. Results: Seventeen studies were included with a total of 1,517,848 patients. A positive association between trauma patient volume and survival was observed in 11/13 studies with adjusted analyses. Few studies addressed other quality domains: efficiency (*n* = 5), safety (*n* = 2), and time aspects of care (*n* = 4). None covered people-centeredness, equitability, or integrated care. Conclusions: Most studies showed a better survival of trauma patients when treated in high-volume hospitals compared to lower volume hospitals. However, the ideal threshold could not be determined. The association between trauma volume and other domains of the quality of healthcare remains unclear.

## 1. Introduction

Trauma care in the Netherlands has evolved over the last two decades through changes on an organizational level as well as changes to the approach of treating trauma as a distinct medical condition. Notably, the central region of the country has observed a decrease in in-hospital mortality rates for severe trauma patients with an Injury Severity Score (ISS) > 15. The mortality rated dropped from 7.9% during 1996–1998 to 5.2% in 2014–2016 [1].

In 2020, the Dutch National Trauma Registration recorded data from 71,623 patients who were urgently admitted due to injuries across 86 hospitals. Among these admissions, 8% involved severely injured patients (Injury Severity Score (ISS) > 15). The initial care for 70% of these seriously injured patients takes place at one of the eleven level 1 Major Trauma Centers (MTCs), spread across thirteen locations. The annual volume of severe trauma cases (ISS > 15) varies between 76 and 452 patients per trauma center location [2].

### 1.1. Healthcare Challenges

Healthcare expenses in the Netherlands surged by 65% between 2000 and 2019, and they are projected to nearly double to 192 billion euros in 2040 [3]. A growing shortage of healthcare workers, exacerbated by the aging population, underscores the need for changes to ensure accessible, affordable, and high-quality healthcare [4]. In response, key healthcare stakeholders, including the government, hospitals, primary care physicians, and long-term care organizations, reached a landmark agreement in 2022, known as the Integrated Care Agreement (ICA, in Dutch: Integraal Zorgakkoord). Among others, this agreement prioritizes timely and suitable care, including for trauma patients. Notably, the ICA mandates that 90% of severe trauma patients (ISS > 15) must be directly admitted to a designated MTC within the trauma region. Each MTC should manage at least 240 trauma patients with an ISS > 15 annually [4]. These criteria for level 1 MTC align with the standards set by the American College of Surgeons [5]. Consequently, several existing MTCs in the Netherlands will need to either shut down or merge to meet this goal.

### 1.2. Trauma Patient Volume and Quality of Healthcare

Centralization of severe trauma patients boosts patient volumes at MTCs for more specialized care. The World Health Organization (WHO) identifies seven dimensions of healthcare quality: effectivity, safety, patient-centeredness, timeliness, equitability, integration, and efficiency (Box 1) [6]. Sewalt et al. prior systematic review and meta-analysis involved 18 articles revealing a modest link between high-volume centers and lower mortality rates in severely injured patients [7]. Their analysis supports that an annual admission of at least 240 severe trauma patients is associated with lower mortality. However, the association between trauma patient volume and other quality domains remains unexplored by a systematic review.

Box 1 **Quality of healthcare according to the World Health Organization [6].**

*“Quality healthcare can be defined in many ways but there is growing acknowledgement that quality health services should be:*

- *Effective—providing evidence-based healthcare services to those who need them;*
- *Safe—avoiding harm to people for whom the care is intended; and*
- *People-centered—providing care that responds to individual preferences, needs and values.*

*To realize the benefits of quality health care, health services must be:*

- *Timely—reducing waiting times and sometimes harmful delays;*
- *Equitable—providing care that does not vary in quality on account of gender, ethnicity, geographic location, and socio-economic status;*
- *Integrated—providing care that makes available the full range of health services throughout the life course;*
- *Efficient—maximizing the benefit of available resources and avoiding waste.”*

Understanding how trauma patient volume affects healthcare quality is crucial for making informed decisions about centralizing trauma care. This review aims to summarize evidence published during the last decade on the association between trauma patient volume and different aspects of healthcare quality.

## 2. Materials and Methods

The conduct of this scoping review was guided and reported according to the Preferred Reporting Items for Systematic Reviews and Meta-Analyses Extension for Scoping Reviews (PRISMA-ScR) [8]. This review has not been registered in a database.

### 2.1. Literature Search

PubMed was searched until 1 August 2023 for relevant publications in the last ten years. Search queries included synonyms and MeSH terms for ‘Advanced Trauma Life Support Care’ or ‘Multiple Trauma’ and ‘centralized hospital service’ or ‘volume’. The complete search strategy is provided in Table 1. References of included studies were checked for relevant publications.

### 2.2. Study Selection

Titles and abstracts were screened independently by two authors (Y.C. and W.F.). Disagreements and discrepancies between the two authors were resolved by a third author (D.F.). Studies were eligible for retrieval when reporting trauma patient volume in relation to at least one quality aspect of healthcare, as defined by the World Health Organization [6]. We excluded systematic reviews, case reports, non-English language abstracts, and studies not evaluating volume–outcome associations.

### 2.3. Data Extraction and Data Analysis

Data extraction was performed independently by three authors (Y.C., D.F., and W.F.) using a data extraction form. Discrepancies and disagreements were discussed until a consensus was reached. Extracted data were study design, location (country), sample size, population, summary statistics of age, volume definition, and outcomes of adjusted analyses (including adjusted factors) and univariate (unadjusted) analyses. Authors of included studies were not contacted for additional data. A quality assessment of included studies was performed according to the previous literature by two authors (D.F and W.F.) [7,9].

Outcomes of included studies were categorized according to the quality aspects of healthcare. Results on mortality and survival were categorized as outcomes regarding the ‘effectivity’, as this is one of the aims of trauma care. The number of studies on volume–outcome associations for the different quality aspects of care were visualized by bar charts. Data were not pooled because of the broad literature search and varying study populations.

## 3. Results

**Figure 1:**
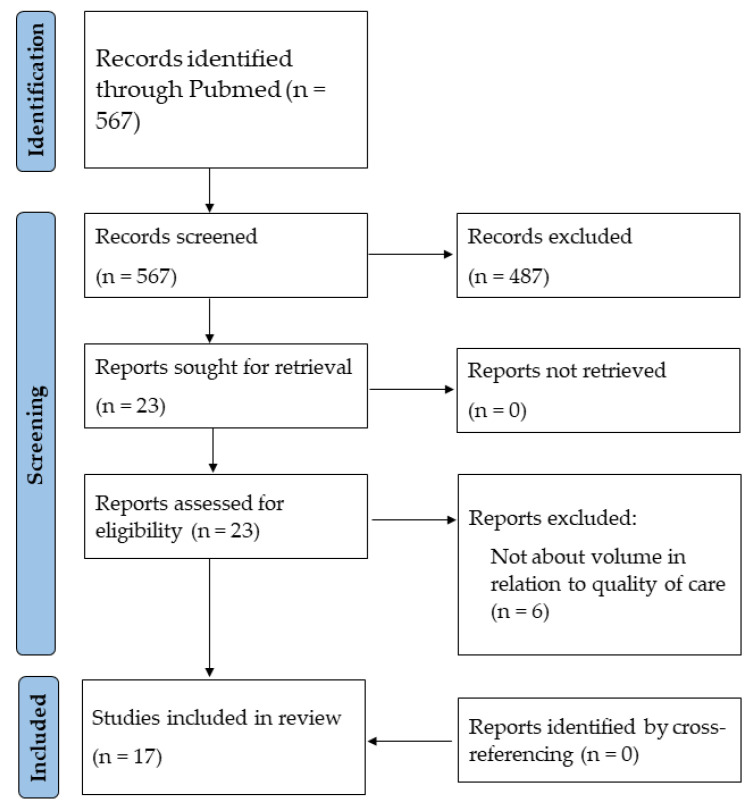
PRISMA flowchart of study selection.

Our search identified a total of 567 studies. After screening titles and abstracts, 23 studies were assessed for eligibility, of which six studies were excluded because these studies did not evaluate volume–outcome associations (Figure 1). Finally, 17 studies were included in this review with a total sample size of 1,517,848 patients. Studies were conducted in the United States (*n* = 8), Japan (*n* = 5), Germany (*n* = 2), England and Wales (*n* = 1), and the Netherlands (*n* = 1). Study characteristics of included studies were summarized in Table 2.

All studies specified their research period, eligibility criteria, and volume definitions. National trauma databanks were used in 15 studies and statewide databanks in 2 studies. Among the studied volume-outcome associations, 18 analyses were adjusted for patient demographics and injury severity (Table 3). Of the 17 studies included, 16 explored the volume’s impact on effectivity (mortality) [10,11,12,13,14,15,16,17,18,19,20,21,22,23,24,25], 5 on efficiency [10,13,15,20,23], 2 on safety [11,23], and 4 on timeliness [20,21,23,26].

### 3.1. Effectivity

**Figure 2:**
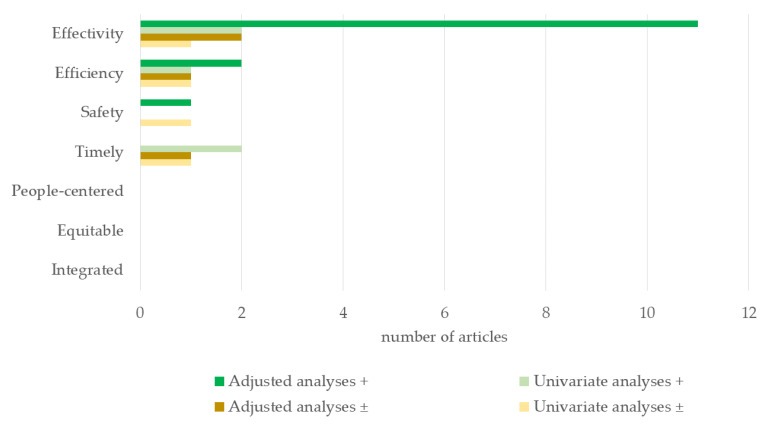
Number of publications on trauma patient volume and quality of care. + = Adjusted or univariate analyses in favor of high volume of trauma patients; ± = no association between trauma patient volume and the evaluated quality aspect of healthcare.

Sixteen included studies reported on mortality or survival, of which results are summarized in Table 4 and Figure 2. Eight studies included only severe trauma patients (ISS > 15 or comparable) [12,14,15,16,20,21,22,24], two studies included only geriatric trauma patients [11,13], and two other studies included only patients with penetrating injuries [19,25]. The other studies reported on the effects of increased volumes of severe and non-severe trauma patients combined (ISS > 15 and ISS ≤ 15; *n* = 1) [17], or specific body parts (*n* = 3) [10,18,23].

Thirteen studies evaluated mortality or survival by analyses adjusting for confounders (e.g., trauma severity, age, and gender). Eleven studies observed a significantly better survival in favor of hospitals with higher trauma volumes [10,11,12,13,15,16,17,18,19,21,23].

For severe trauma patients specifically, four out of six studies with adjusted analyses observed a positive association between trauma patient volume and survival [12,13,15,21]. Zacher et al. observed that severe trauma patient volume was significantly associated with survival in Germany (OR 1.001 per patient per year, *p* = 0.01) [12]. Aoki et al. observed that the high volume of severe trauma patients was associated with reduced in-hospital mortality after correcting for confounders in Japan (adjusted OR = 0.757, 95%CI 0.626–0.916 [21]). Endo et al. showed that the severe trauma patient volume was significantly associated with higher in-hospital survival for each 50-patient increase in Japan (adjusted OR 1.16, 95%CI 1.12–1.21) [10]. Moreover, per 100 increase in severe geriatric trauma patients, Olufajo et al. observed a significant decrease in hospital mortality in the United States (adjusted OR 0.89; CI 0.82–0.97) [13]. Two studies with adjusted analyses did not observe an association between severe trauma patient volume and survival/mortality [20,22]. Sewalt et al. did not find an association between volume (1st tertile ≤ 163 vs. 3rd tertile > 191 annual severe trauma patients) and mortality in English MTCs in their analyses, adjusting for 10 possible confounders [20]. Likewise, Sewalt et al. did not find an association between volume and mortality in MTCs in The Netherlands [22].

Both studies on geriatric trauma performed adjusted analyses and showed a positive association between geriatric trauma patient volume and survival in the United States [11,13]. A pediatric trauma population was studied by Floan et al., who observed that a higher annual case volume was associated with lower observed mortality compared to the expected mortality for penetrating firearm-related thoracic traumas in pediatric patients in the United States. No such association was observed for cut/pierce trauma [25].

Two other studies evaluated the association of trauma volume and mortality or survival in univariate analyses with varying results [14,24]; these results are summarized in Table 4.

### 3.2. Efficiency

Five studies reported on the efficiency aspects of healthcare in relation to trauma volume (Table 5, Figure 2). Adjusted analyses were reported in three studies [10,15,20]. Sewalt et al. observed a significant association between the volume of severe trauma patients and shorter critical care length of stay for each 10-patient increase (adjusted OR 0.47, 95%CI 0.02–0.94) [20]. In another study, increased severe trauma patient volume was significantly associated with lower total costs per admission for each 50-patient increase (adjusted difference −$488.0 (95%CI −$818.0 to −$158.0) [15]. However, Clement et al. did not observe a significant difference in average cost per patient with neurological trauma between the hospitals with <6 cases/year as compared with hospitals with higher volumes [10].

Two studies performed univariate analyses evaluating the relation between trauma volume and length of stay with varying results [23,25] (Table 5).

### 3.3. Safety

Two studies reported on the safety aspects of healthcare in relation to trauma volume [11,23] (Figure 2). Matsushima et al. adjusted for patient characteristics, injury severity, and comorbidities. They showed that for a 100-patient increase in the annual volume of geriatric trauma, the risk of major complications is significantly lower (adjusted OR 0.79, 95%CI 0.63–0.99). In addition, there is a lower risk of failure to rescue in cases where a major complication occurs [11]. The study by Tang et al. evaluated the association between low, medium, and high volumes of emergent laparotomies for hemorrhage control and major complications. In a univariate analysis, no association was observed between volumes of emergent laparotomies and major complications [23].

### 3.4. Timeliness

Four studies reported on the time-related aspects of healthcare in relation to trauma volume [20,21,23,26]. Sewalt et al. was the only study evaluating the results on time in adjusted analyses. No association between hospital volume and time to computed tomography or time to operation was observed in England [20].

Two studies performed univariate analyses showing that an increased hospital volume was associated with decreased door-to-definitive treatment time of severe trauma patients and time to laparotomy of severe trauma patients [21,23] (Figure 2). Although transportation times to large hospitals were longer in a German study by Lefering et al., a more efficient workflow (including shorter times to diagnostic procedures) at the emergency department resulted in an overall ~10 min faster time from the accident to the end of the emergency department treatment. However, formal statistical analyses were not performed [26].

### 3.5. People-Centred, Equitable, and Integrated Qualities

None of the included studies reported specifically on the quality aspects of being ‘people-centered’, ‘equitable’, or ‘integrated’.

## 4. Discussion

### 4.1. Summary of Evidence

Reviews investigating the association between high-volume trauma centers and quality of healthcare are scarce. This scoping review showed a better survival of trauma patients in high-volume trauma centers compared to lower volume centers. However, trauma volume definitions varied among studies. There was little to no evidence observed demonstrating that a higher volume of trauma patients was associated with better quality in the other quality domains of healthcare.

Currently, the centralization of trauma care and trauma patient volume is an important topic for MTC in the Netherlands. The ICA requires a minimum of 240 patients with an ISS > 15 annually for MTCs [4]. Subsequently, this threshold will have consequences for several MTCs in the Netherlands. In this review, 11/13 included studies with adjusted analyses showed better survival in high-volume centers compared to low-volume centers. For severely injured patients specifically, 4/6 studies observed such positive associating between trauma patient volume and survival. None of the centers with high trauma patient volume showed higher mortality compared to centers with lower trauma patient volume. High-volume trauma centers have, by definition, more experience in trauma care, which may be associated with process optimization in the initial acute care for trauma patients in the Emergency Room, and within the hospital after the initial assessment and treatment. With increasing volumes, trauma care may benefit from defined healthcare paths and established multidisciplinary collaborations, which may improve the survival of trauma patients with an ISS > 15.

### 4.2. Strengths and Limitations

This scoping review evaluated available evidence of the effects of centralization and higher volumes of trauma care on all quality aspects of healthcare according to the WHO. The study was performed according to the PRISMA-ScR in a standardized matter.

Limitations of this study should be acknowledged. First, only articles published in the last 10 years were included in our review. Articles about the association between trauma patient volume and survival (effectivity) published longer ago have already been evaluated by Caputo et al. in 2014 and Sewalt et al. in 2018 [7,27]. However, studies before 2013 may have evaluated the effects of centralization and hospital volume on different aspects of the quality of care for trauma patients. These studies were not included in the present review.

Second, the included studies in this review showed large differences in study populations and volume definitions. Most studies showed that high-volume trauma centers were associated with a better survival. In order to provide quantitative summary estimated, results of studies may be pooled. However, a pooling of the results from various studies on mortality was not possible in this review due to the heterogeneity of studies. These findings and conclusions are in line with the findings from the previous review by Caputo et al. [27]. As a result, the ideal threshold of trauma patient volume per center could not be determined.

Third, regarding mortality analyses, 10/13 studies with adjusted data were conducted in the United States and Japan, consistently showing a positive link between patient volume and survival. However, applying these findings to Europe, particularly the Netherlands, may pose challenges. Among the European-based studies (*n* = 3), only one indicated a positive volume-survival association. In-hospital mortality/survival can be influenced by transportation time to MTCs, as severely injured patients may potentially decease during transportation before arrival at an MTC. Longer transportation times might lead to better in-hospital survival for MTC when transportation times are long. Most studies on in-hospital mortality did not adjust for transportation time. Nevertheless, Lefering et al. noted longer travel times to large German hospitals but a more efficient workflow at the emergency department, resulting in an overall ~10 min faster time from the accident to treatment completion [26]. Additionally, transportation time’s impact may vary more in countries with extensive distances, whereas smaller countries with ample MTCs might experience fewer challenges.

Fourth, mortality/survival (‘effectiveness’) is only one of the seven aspects of quality care indicators. Safety, timeliness, and efficiency were only in a few studies investigated. The quality domains ‘people-centered, equitable and integrated’ are notably entirely absent in the included studies. The effects of centralization and trauma patients’ volume on the other quality domains of healthcare are important topics to be evaluated in future studies.

### 4.3. Implications and Next Steps for Trauma Care in the Netherlands

The ICA states a minimum of 240 severely injured patients (ISS > 15) per MTC location per year. Based on numbers from the 2016–2020 report from the Dutch trauma registry, annual ISS > 15 patients were <240 in six MTC locations, 240–300 in four MTC locations, and >300 in three MTC locations [2]. As a result, the six centers not fulfilling the criteria of 240 annual ISS > 15 patients either need to merge, treat more patients, or stop as an MTC. The formerly two MTC locations in Amsterdam continue in one location, as well as the two MTC locations in The Hague [28,29]. This centralization will result in higher volumes (>240) of severely injured patients in these locations. The two remaining centers with <240 annual severe trauma patient may treat more patients to some extent by improving the prehospital triage.

In the Netherlands, 35% of severely injured patients are not initially treated at an MTC [30]. The optimization of prehospital triage is needed to reach the set target of >90% of severe trauma patients that is directly transported to a MTC. Dutch data showed that the optimization of pre-hospital triage may lower the under-triage to 11% with an over-triage of 50% [31]. An optimal prehospital triage therefore would result in the further centralization of severe trauma patients. However, a pre-hospital triage is performed in the heat of the moment without all facilities of an MTC, which makes a complete assessment impossible. On the contrary, the ISS is defined retrospectively as a measure of trauma severity based on all injuries, of which many are detected in-hospital using available diagnostics, such as computed tomography imaging. Studies using a smartphone application with a prediction model that help emergency medical services in the triage of trauma patients have been initiated [32]. Recent data suggests that such smartphone applications may lower the under-triage from 32% to 27% (adjusted OR for under-triage of 0.78; 95%CI: 0.63–0.96) without an increase in over-triage [33]. However, further work is required to enhance the prehospital triage for the direct transfer of severely injured patients to MTCs. In 2020, the 30-day mortality rate for Dutch trauma patients was 5% [2]. The impact of centralizing severely injured patients by requiring a minimum of 240 cases annually per MTC and striving to transfer over 90% of such patients to MTCs is yet to be monitored.

Due to the ICA, trauma care in the Netherlands will be more centralized, with increased volumes of severely injured patients per center. This could necessitate adjustments in the existing MTC locations to accommodate the anticipated rise in severe trauma patients. This might involve additional trained healthcare staff and improved facilities in the emergency department, wards, and supporting specialties.

With higher severe trauma patient volumes in these MTC, more experience in specific trauma patient subgroups may lead to the identification of special needs for those subgroups (e.g., for geriatric trauma). Trauma care involves multiple healthcare disciplines; finding the right balance between concentrating care and maintaining trauma surgeons’ expertise on specific trauma profiles is crucial for optimizing patient outcomes and sustaining professional engagement. However, trauma care is not only about survival. Quality of life is what counts for the patient in the end. After surviving major injuries, patients may fully recover, need rehabilitation, or have to make (major) adjustments in their life because of permanent disabilities as a result of their injuries. Therefore, future studies may include outcomes on the quality of life and other patient-reported outcomes to gain more insights into all aspects of trauma care.

## 5. Conclusions

The majority of included studies showed a better survival of trauma patients in high-volume hospitals compared to lower volume hospitals. Included studies were heterogeneous in trauma populations, study design, and analyses. As a result, a pooling of results was not possible and the ideal threshold of trauma patient volume/center could not be exactly determined. The evidence on the effects of centralization and the high volume of trauma care in the other six domains of the quality of healthcare is scarce or absent. This remains to be evaluated in future studies, as these aspects become important when patients survive major injuries.

## Figures and Tables

**Table 1:** Literature search queries.

PubMed	
1.	(Advanced Trauma Life Support Care[MeSH Terms] OR Multiple Trauma[MeSH Terms] OR polytrauma[Title/Abstract] OR multitrauma[Title/Abstract] OR trauma care[Title/Abstract] OR trauma patient[Title/Abstract] OR trauma victim[Title/Abstract] OR severely injured[Title/Abstract])
2.	(Centralized hospital service[MeSH Terms] OR centralized[Title/Abstract] OR decentralized[Title/Abstract] OR centralization[Title/Abstract] OR volume[Title/Abstract])
3.	(y_10[Filter])
4.	1 AND 2 AND 3

**Table 2:** Study characteristics.

Study	Design	Country	Sample Size (N)	Population	Age (Years)	Volume Definition
Clement et al., 2013 [10]	Retrospective, multicenter (Nationwide Inpatient Sample 2006)	United States	12,493	Patients with neurological trauma (ICD-9 codes for subarachnoid, subdural, and extradural hematoma)	Range: 15–85+	Categories: <6 [reference], 6–11, 12–23, 24–59, and 60+ annual patients with neurological trauma
Matsushima et al., 2014 [11]	Retrospective, multicenter (Statewide trauma registry 2001–2010)	United States	39,431	Geriatric trauma patients, median ISS = 13; 43% with ISS > 15	Median 80 (range: 66–114)	Analyses for 100-patient increase in annual volume
Zacher et al., 2015 [12]	Retrospective, multicenter (TraumaRegister DGU 2009–2013)	Germany	39,289	Severe trauma patients with ISS > 15, mean ISS = 27 (sd ± 12)	Mean: 50 (sd ± 22)	Continuous
Olufajo et al., 2016 [13]	Retrospective, multicenter (California State Inpatient Database 2007–2011)	United States	61,915	Geriatric trauma patients >65 years	65–84: 67% >85: 33%	Analyses for 100-patient increase in annual volume
Brown et al., 2017 [14]	Retrospective, multicenter (National trauma data bank 2000–2012)	United States	839,809	Severe trauma patients with ISS > 15	Median: 41 (IQR 23–60)	Categories based on median: ≤247, >247 annual severe trauma patients
Endo et al., 2017 [15]	Retrospective, multicenter (National trauma data bank 2010–2015)	Japan	116,329	Severe trauma patients ≥16 years (ICD-10 based trauma severity scoring system)	Median: 67–75	Categories: 1–50 [reference], 51–100, 101–150, 151–200, and >200 annual severe trauma patients ≥16 years
Miyata et al., 2017 [16]	Retrospective, multicenter (National trauma data bank 2007–2014)	United States	3747	Severely injured pediatric patients with ISS > 15 treated at adult trauma centers	10–15: 64%	Quartiles: 8–93 [reference], 94–179, 180–265, and 266–352 annual severe pediatric trauma patients
Nakahara et al., 2017 [17]	Retrospective, multicenter (National trauma data bank 2012–2013)	Japan	12,378	Blunt trauma patients ≥15 years and ISS ≥ 9 (49.9% ISS > 15)	Categories: 15–24: 10% 25–44: 16% 45–64: 24% ≥65: 50%	Quartiles: ≤124 [reference], 125–178, 179–312, and ≥313 annual blunt trauma patients ≥15 years and ISS ≥ 9
Wada et al., 2018 [18]	Retrospective, multicenter (National trauma data bank 2010–2014)	Japan	7725	Severe torso injury patients with emergency surgery or interventional radiology treatment	Median 58–61	Quartiles: ≤8 [reference], 8- ≤ 13, 13- ≤ 20, and >20 annual trauma patients with ISS > 15
Fu et al., 2019 [19]	Retrospective, multicenter (National trauma data bank 2011–2015)	United States	55,696	Penetrating injury patients; 18% with ISS > 15	Mean 33–35	Quartiles: ≤37 (bottom 25%) [reference] vs. ≥167 (top 25%) annual penetrating injury patients
Sewalt et al., 2020 [20]	Retrospective, multicenter (National trauma data bank 2013–2016)	England and Wales	47,159	All trauma patients with ISS > 15 in major trauma center	Median 53 (IQR 32–74)	Tertiles: ≤163, 164–191, and >191 annual trauma patients with an ISS > 15
Aoki et al., 2021 [21]	Retrospective, multicenter (National trauma data bank 2004–2015)	Japan	74,957	Severe trauma patients with ISS > 15	Median: 60 (IQR 38–74)	Categories: LV 1–49, MV 50–99, and HV ≥ 100 annual trauma patients with ISS > 15
Sewalt et al., 2021 [22]	Retrospective, multicenter (Dutch Trauma registry 2015–2018)	The Netherlands	11,917	Severe adult trauma patients with ISS > 15 admitted to a MTC	LV: median 58 (IQR 35–74) HV: median 53 (IQR 30–69)	Analyses for 50-patient increase in annual volume
Tang et al., 2021 [23]	Retrospective, multicenter (Trauma Quality Improvement Program database 2017)	United States	8588	Blunt and penetrating trauma patients with emergent laparotomies < 24 h for hemorrhage control	Blunt, penetrating: 18–44: 52%, 79% 45–64: 32%, 18% ≥65: 16%, 3%	Categories: LV ≤ 12, MV 13–24, and HV ≥ 25 annual emergent laparotomies for hemorrhage control
Toida et al., 2021 [24]	Retrospective, multicenter (National trauma data bank 2014–2018)	Japan	53,088	Severe trauma patients with ISS >15	Range 0–65+	Categories: LV < 150 annual trauma patients with ISS > 15, and HV ≥ 150
Floan et al., 2022 [25]	Retrospective, multicenter (National trauma data bank 2013–2016)	United States	4134	Pediatric penetrating thoracic trauma	Mean 15 (sd ± 3.5)	Continuous
Lefering et al., 2022 [26]	Retrospective, multicenter (Trauma-Register DGU 2013–2017)	Germany	129,193	Trauma patients, mean ISS 18 (sd ± 12), 50% with ISS > 15	Mean 51 (sd ± 22)	Categories: 1–9, 10–19, 20–39, 40–99, 100+ annual trauma patients

Abbreviations: HV = high volume; ISS = Injury Severity Score; LV = low volume; MV = medium volume; IQR = interquartile range; sd = standard deviation.

**Table 3:** Quality assessment.

Study	Nationwide (Sample)	Type of Hospitals Reported	Time Period Reported	Eligibility Criteria Reported	Volume Definitions Reported	N Severely Injured Patients Reported	Reported Quality Aspect of Healthcare	Volume-Outcome: Odds Ratio or Absolute Values	95%CI or *p*-Value Reported	Analyses Adjusted for Patient Demographics	Analyses Adjusted for Injury Severity	Analyses Adjusted for Trauma-Center Level	Funding Sources Reported or No Conflicts of Interest
Clement et al., 2013 [10]	+	+	+	+	+	−	Effectivity Efficiency	+ +	+ +	+ +	+ +	− −	+
Matsushima et al., 2014 [11]	−	+	+	+	+	+	Effectivity Safety	+ +	+ +	+ +	+ +	− −	+
Zacher et al., 2015 [12]	+	+	+	+	+	+	Effectivity	+	+	+ *	+ *	+	+
Olufajo et al., 2016 [13]	−	+	+	+	+	+	Effectivity Efficiency	+ +	+ +	+ +	+ +	+ +	+
Brown et al., 2017 [14]	+	−	+	+	+	+	Effectivity	+	+	~ ^1^	~ ^1^	−	+
Endo et al., 2017 [15]	+	+	+	+	+	+	Effectivity Efficiency	+ +	+ +	− −	+ +	− −	+
Miyata et al., 2017 [16]	+	+	+	+	+	+	Effectivity	+	+	+	+	+	−
Nakahara et al., 2017 [17]	+	+	+	+	+	+	Effectivity	+	+	+	+	−	+
Wada et al., 2018 [18]	+	+	+	+	+	+	Effectivity	+	+	+	+	−	+
Fu et al., 2019 [19]	+	+	+	+	+	+	Effectivity	+	+	+	+	−	+
Sewalt et al., 2020 [20]	+	+	+	+	+	+	Effectivity Efficiency Timely	+ + +	+ + +	+ + +	+ + +	NA NA NA	+
Aoki et al., 2021 [21]	+	−	+	+	+	+	Effectivity Timely	+ +	+ +	+ −	+ −	− −	+
Sewalt et al., 2021 [22]	+	+	+	+	+	+	Effectivity	+	+	+	+	NA	+
Tang et al., 2021 [23]	+	+	+	+	+	+	Effectivity Efficiency Safety Timely	+ + + +	+ + + +	+ − − −	+ − − −	+ − − −	+
Toida et al., 2021 [24]	+	+	+	+	+	+	Effectivity	+	+	−	−	−	+
Floan et al., 2022 [25]	+	+	+	+	+	−	Effectivity	−	+	−	~ ^2^	−	+
Lefering et al., 2022 [26]	+	−	+	+	+	+	Timely	+	−	−	−	−	+

Abbreviations: + = assessment criteria positive, − = assessment criteria negative. CI = confidence interval; N = number; NA = not applicable, as study included major trauma centers only; * = adjusted for a death prediction score taking patient demographics and worst and second worst injuries into account. ~ ^1^ = center-level standardized mortality ratios based on, e.g., patients’ characteristics and injury severity, were used in outcome analyses. ~ ^2^ = hospital performance based on observed mortality and expected mortality according to a trauma mortality prediction model.

**Table 4:** Effectivity.

Study	Outcome (Adjusted)	
United States		
Clement et al., 2013 [10]	Hospitals with 6+ cases/year with subarachnoid, subdural, and extradural hematomas had significant lower mortality rates compared to hospitals with <6 annual cases (adjusted ORs range 0.45–0.63). Adjusted for, e.g., age, sex, geographical region, hospital characteristics, comorbidities, other severe head trauma, neurosurgical procedures performed, significant non-neurological injury, and severity of intracranial hemorrhage	+
Matsushima et al., 2014 [11]	Larger institutional volume of geriatric trauma cases was significantly associated with lower in-hospital mortality (adjusted OR 0.75 for a 100-patient increase; CI 0.61–0.92). Adjusted for patient characteristics, injury severity, and preexisting conditions	+
Olufajo et al., 2016 [13]	Significant decrease in hospital mortality per 100 increase in geriatric trauma patients with ISS > 15 (adjusted OR 0.89; CI 0.82–0.97). Adjusted for patient demographic, injury, admission, and hospital characteristics	+
Miyata et al., 2017 [16]	Highest volume group was associated with lower mortality compared to the lowest quartile volume center (adjusted OR 0.47; CI 0.30–0.75; *p* < 0.01). For level 1 trauma centers specifically, the highest volume group was associated with lower mortality compared to the lowest quartile volume center (adjusted OR 0.50; CI 0.31–0.79; *p* < 0.01). Adjusted for trauma center characteristics and patient characteristics (e.g., age, injury severity)	+
Fu et al., 2019 [19]	Significant increase in survival per 10 increase in penetrating injury patients (adjusted OR 1.01, *p* = 0.03). Adjusted for age, pulse, systolic blood pressure, ventilation, ISS and total number of trauma patients	+
Tang et al., 2021 [23]	Lower odds of in-hospital mortality in HV centers for blunt injury patients (adjusted OR 0.74; CI 0.59–0.93; *p* = 0.011) and penetrating injury patients (adjusted 0.86; CI 0.77–0.96; *p* = 0.023) with emergent laparotomies < 24 h for hemorrhage control. Adjusted for age, sex, comorbidities, systolic blood pressure, GCS, prehospital cardiac arrest, ISS, trauma center level, and injury-specific center laparotomy volume	+
Japan		
Endo et al., 2017 [15]	Severe trauma patient volume was significantly associated with higher in-hospital survival for each 50-patient increase (adjusted OR 1.16; CI 1.12–1.21). Adjusted for trauma severity and hospital characteristics	+
Nakahara et al., 2017 [17]	Higher patient volume was significantly associated with lower 30-day mortality risk (HR for the highest vs. lowest quartile (adjusted OR 0.74; CI 0.56–0.98). Adjusted for age, gender, GCS, blood pressure, respiratory rate, and ISS	+
Wada et al., 2018 [18]	Hospitals with >20 patients with severe torso injuries had a significant lower 1-d mortality (adjusted OR 0.64; CI 0.43–0.96) and 28-d mortality (adjusted OR 0.59; CI 0.44–0.79) compared to hospitals with <8 patients with severe torso injuries. Adjusted for age, gender, rural hospital, Japan Coma Score, trauma severity, mechanical ventilation on admission, transfusion on admission, and neurosurgery on admission	+
Aoki et al., 2020 [21]	Severe trauma patient (ISS > 15) volume was associated with reduced in-hospital mortality compared to low volume (adjusted OR = 0.76; CI = 0.63–0.92). Adjusted for age, gender, cause of injury, vital signs, ISS, and hospital	+
Europe		
Zacher et al., 2015[12]	Severe trauma patient volume was significantly associated with survival in Germany (OR 1.001 per patient per year (for each patient increase); *p* = 0.01). Adjusted for Revised Injury Severity Classification (RISC) II score, number of patients per year, and hospital level of care	+
Sewalt et al., 2020 [20]	No association between hospital volume and in-hospital mortality of severe trauma patients in England with ISS > 15 (adjusted OR 1.02; CI 0.68–1.54; *p* = 0.92). Adjusted for age, gender, ISS, Revised Trauma Score, comorbidities, penetrating injury, Abbreviated Injury Score, head injury, and referral	±
Sewalt et al., 2021 [22]	No association between hospital volume and in-hospital mortality in the Netherlands (OR 0.97 per 50 extra patients; CI 0.90–1.04, *p* = 0.44). Adjusted for age, sex, ISS, systolic blood pressure, respiratory rate, GCS, prehospital intubation, ASA, penetrating injury, and Abbreviated Injury Score for head injury	±
	Outcome (Unadjusted)	
Brown et al., 2017 [14]	Each 1% increase in volume was associated with 73% increased odds of improving standardized mortality ratios over time in the United States (OR 1.73; CI 1.03–2.91; *p* = 0.03). Standardized mortality rates included age, several clinical parameters at admission, GCS, ISS, and mechanism of injury	+
Toida et al., 2021 [24]	No significant differences in in-hospital mortality for severe trauma patients (ISS > 15) in Japan between high-volume and low-volume hospitals (median 2.13% vs. 0%), *p* = 0.25)	±
Floan et al., 2022 [25]	Higher annual case volume was associated with improved hospital performance (lower observed mortality compared to expected mortality) for firearm-related thoracic trauma in pediatric patients in the United States, but not for cut/pierce trauma	+

Abbreviations: CI = 95% confidence interval; GCS = Glasgow Coma Scale; HV = high volume; ISS = Injury Severity Score; OR = odds ratio. + = Adjusted or univariate analyses in favor of high volume of trauma patients/centralization of trauma care; ± = no association between trauma patient volume and the evaluated quality aspect of healthcare.

**Table 5:** Efficiency.

Study	Outcome (Adjusted)	
Clement et al., 2013 [10]	No significant difference in the average cost per case with subarachnoid, subdural, and extradural hematoma between the hospital cohort <6 cases/year as compared with hospitals with more cases annually. Adjusted for, e.g., age, sex, geographical region, hospital characteristics, comorbidities, other severe head trauma, neurosurgical procedures performed, significant non-neurological injury, and severity of intracranial hemorrhage	±
Endo et al., 2017 [15]	Increased severe trauma patient volume was significantly associated with lower total costs per admission for each 50-patient increase (adjusted difference −$488.0 (CI −$818.0 to −$158.0)). Adjusted for trauma severity and hospital characteristics	+
Sewalt et al., 2020 [20]	Significant association between hospital volume and critical care length of stay for each 10-patient with (ISS > 15) increase (adjusted OR 0.47; CI 0.02–0.94). Adjusted for age, gender, ISS, Revised Trauma Score, comorbidities, penetrating injury, Abbreviated Injury Score, head injury and referral	+
	**Outcome (Unadjusted)**	
Tang et al., 2021 [23]	No difference between high-, medium-, and low-volume hospitals in hospital length of stay for blunt and penetrating trauma patients with emergent laparotomies <24 h for hemorrhage control	±
Floan et al., 2022 [25]	A higher annual case volume of pediatric penetrating thoracic trauma was associated with significant shorter hospital and intensive care length of stay	+

Abbreviations: CI = 95% confidence interval; OR = odds ratio. + = Adjusted or univariate analyses in favor of high volume of trauma patients/centralization of trauma care; ± = no association between trauma patient volume and the evaluated quality aspect of healthcare.

## Data Availability

No new data were created or analyzed in this study. Data sharing is not applicable to this article.
